# Evaluation of Dry Eye and Meibomian Gland Dysfunction in Teenagers with Myopia through Noninvasive Keratograph

**DOI:** 10.1155/2016/6761206

**Published:** 2016-01-06

**Authors:** Xiu Wang, Xiaoxiao Lu, Jun Yang, Ruihua Wei, Liyuan Yang, Shaozhen Zhao, Xilian Wang

**Affiliations:** ^1^Tianjin Medical University Eye Hospital, Fukang Road No. 251, Nankai District, Tianjin 300384, China; ^2^Tianjin Beichen Hospital, Beiyi Road No. 7, Beichen District, Tianjin 300400, China

## Abstract

*Purpose*. This study aims to evaluate dry eye and ocular surface conditions of myopic teenagers by using questionnaire and clinical examinations.* Methods*. A total of 496 eyes from 248 myopic teenagers (7–18 years old) were studied. We administered Ocular Surface Disease Index (OSDI) questionnaire, slit-lamp examination, and Keratograph 5M. The patients were divided into 2 groups based on OSDI dry eye standard, and their ocular surfaces and meibomian gland conditions were evaluated.* Results*. The tear meniscus heights of the dry eye and normal groups were in normal range. Corneal fluorescein scores were significantly higher whereas noninvasive break-up time was dramatically shorter in the dry eye group than in the normal group. All three meibomian gland dysfunction parameters (i.e., meibomian gland orifice scores, meibomian gland secretion scores, and meibomian gland dropout scores) of the dry eye group were significantly higher than those of the normal group (*P* < 0.0001).* Conclusions*. The prevalence of dry eye in myopic teenagers is 18.95%. Meibomian gland dysfunction plays an important role in dry eye in myopic teenagers. The Keratograph 5M appears to provide an effective noninvasive method for assessing ocular surface situation of myopic teenagers.

## 1. Introduction

Dry eye disease is defined by the Report of the Definition and Classification Subcommittee of the International Dry Eye WorkShop as a multifactorial disease of tears and ocular surface, which results in symptoms of discomfort, visual disturbance, and tear film instability, with potential damage to the ocular surface [1]. Dry eye is a common ocular surface disease that often occurs in the elderly [2]. More than 20% of people in 30–40-year-olds have dry eye, and the prevalence of dry eye in people over 70 years old is as high as 36.1% [3]. Currently, with the increasing popularity of computers, video games, and smartphones in the younger generation, the incidence of myopia in teenagers is increasing annually, with a growing number of myopic teenagers exhibiting frequent blinking, sensitivity to light, and other dry eye ocular discomfort [4]. Dry eye is of an increasingly important clinical significance in myopic adolescents as it affects their quality of life. Diagnosis of dry eye currently relies on break-up time (BUT) and Schirmer's tests. However, BUT speed is different for different people. Moreover, fluorescein sodium affects the tear film's stability. BUT and Schirmer's tests are both invasive examinations. Adolescents are more difficult to evaluate than adults for ocular surface dysfunction because of poorer compliance with the procedure. Thus the traditional diagnostic methods for identifying dry eye in adolescents are less definitive since children are more sensitive to the procedure than adults. Accordingly, the data reproducibility is more variable making it more difficult to identify the disease signs in an adolescent population. Accordingly, reported dry eye incidence in myopics is underdiagnosed. Given the lower prevalence of dry eye disease in children, the diagnosis of dry eye is often overlooked by many ophthalmologists [5]. Previous studies have confirmed that Keratograph 5M (Oculus, Wetzlar, Germany) noninvasively measures noninvasive break-up time (NIBUT), tear meniscus height, and meibography with low irritability [6–10]. Therefore, in this study, we used Keratograph 5M combined with slit-lamp examination and dry eye questionnaire to give myopic adolescents a series of dry eye-related inspections and assessments and to determine the prevalence of dry eye and ocular surface conditions among myopic adolescents.

## 2. Materials and Methods

### 2.1. Materials

A total of 248 consecutive patients (average age 12.26 ± 1.86 years, range 7–18 years; 132 female, 116 male, male to female ratio = 1 : 1.14) who went to Tianjin Medical University Eye Hospital myopia clinic from January to June in 2014 with no systemic or ocular treatment, contact lens wear, keratitis, ocular allergic disease, any other ocular surface disease, glaucoma, active and chronic uveitis, or previous ocular surgery or injury were recruited in this prospective study.

Written informed consent was obtained from the parents of the patients. The study was approved by the Institutional Review Board of the Tianjin Medical University Eye Hospital and performed in accordance with the tenets of the Declaration of Helsinki.

### 2.2. Methods

This study was a prospective study, and all inspections were performed by the same experienced examiner.

#### 2.2.1. Questionnaire Regarding Dry Eye

Before clinical examination, each patient completed an Ocular Surface Disease Index (OSDI) questionnaire for assessment of ocular surface symptoms and the severity of dry eye. This questionnaire [11] included questions regarding the frequency of dry eye symptoms experienced in the previous week (light sensitivity, gritty sensation, painful or sore eyes, blurred vision, and poor vision), vision-related daily activities (reading, watching TV, working on computers, and driving at night), and environmental triggers (wind, air conditioning, and low humidity). Each answer was scored on a 5-point scale (all of the time: 4, most of the time: 3, half of the time: 2, some of the time: 1, and none of the time: 0), and the OSDI score was calculated as follows: {(sum of scores × 25)/total number of questions}. Thus, the total OSDI score ranged from 0 to 100. A higher OSDI score represented greater disability. Answering was completed with the assistance of one doctor, and the completion time was controlled within 4–6 min. Currently, no uniform national standards have been established for the diagnosis of dry eye, and the diagnostic criteria are inconsistent worldwide. Based on their OSDI scores, the patients were categorized as having a normal ocular surface (0–12 points) or as having mild (13–22 points), moderate (23–32 points), or severe (33–100 points) ocular surface disease [12]. The study population was divided into normal and dry eye groups, which included those with mild dry eye, moderate dry eye, and severe dry eye. The two groups were compared to assess their ocular surface conditions.

#### 2.2.2. Keratograph 5M: Noninvasive Measurement for Ocular Surface

Keratograph 5M inspection items include noninvasive tear film break-up time, noninvasive tear meniscus height, and meibography. The tests were first measured in the right eye and then the left eye. Three measurements were taken, and the average of results was considered in the statistics.

Keratograph 5M was used to grade the right eyelid using the following meibomian gland dropout degrees as meiboscore [13]: Grade 0: no loss of meibomian gland; Grade 1: loss of < 1/3 of the whole gland area; Grade 2: loss of 1/3-2/3 of the whole gland area; and Grade 3: loss of > 2/3 of the whole gland area. The meiboscore of each eye was calculated as the sum of the scores from both upper and lower eyelids, making the total meiboscore per eye in a range of 0–6.

#### 2.2.3. Slit-Lamp Examination of the Anterior Segment

The following examinations were carried out sequentially using a slit-lamp: meibomian gland orifices, meibomian gland lipid secretion, and corneal fluorescein staining scores.

The quality of the meibomian gland orifices was scored semiquantitatively in the central eight glands of the lower right eyelid as follows: Grade 0 is normal, that is, no obstruction of orifice and being covered with a thin and smooth fluid; Grade 1 is obstruction of one or two meibomian gland orifices or secretions or occlusion; Grade 2 is obstruction of two or three meibomian gland orifices with thick fluid; Grade 3 is obstruction or narrowing of almost half of the meibomian gland orifices; Grade 4 is obstruction or narrowing of more than half of the meibomian gland orifices with sticky secretions.

The quality of the meibum was scored semiquantitatively in the central eight glands of the lower right eyelid as follows (0–24 points in total) [14]: Grade 0: clear fluid; Grade 1: cloudy fluid; Grade 2: cloudy, particulate fluid; and Grade 3: inspissated, toothpaste-like fluid.

Corneal fluorescein staining was graded from 0 to 12, which was a sum of the scores of corneal four quadrants scored individually as 0 (no staining), 1 (mild staining with a few scattered dots of stains), 2 (moderate staining between 1 and 3), and 3 (severe staining with confluent stains or corneal filaments) [15].

### 2.3. Statistical Analysis

Statistical analysis was performed using SPSS version 19.0. All variables were expressed as the mean ± standard deviation. Indexes were analyzed using nonparametric Mann-Whitney *U* test, and the intergroup data were compared using Shapiro-Wilk test. Spearman correlation analysis was used to estimate the correlations between various factors. Categorical variables were compared between the groups using the chi-square test. The confidence interval was set at 95%, and probability values of *P* < 0.05 were considered statically significant.

## 3. Results

### 3.1. Dry Eye Detection Rate

A total of 248 subjects (496 eyes, average age 12.26 ± 1.86 years) were recruited for the study. A total of 116 males (average age 11.9 ± 2.55 years) and 132 females (average age 12.2 ± 2.45 years) participated.

OSDI screened out 201 normal people (81.05%), 23 mild dry eye people (9.27%), 15 moderate dry eye people (6.05%), and 9 severe dry eye people (3.63%). Based on the OSDI dry eye standard, 47 (18.95%) dry eye populations were detected. The right eyes of the 47 dry eye patients were included in the dry eye group (25 males and 22 females) and the right eyes of 201 normal eye patients were included in the normal group (98 males and 103 females). Statistical comparison of the two groups was then carried out.

### 3.2. Comparison of General Condition and Ocular Statistical Indexes between the Dry Eye Group and the Normal Group

Table 1 shows that no significant differences in age, gender, and tear meniscus height were found between the dry eye and the normal groups. Tear meniscus height was normal for both groups (>0.20 mm), with 0.23 ± 0.03 mm in the dry eye group and 0.22 ± 0.03 mm in the normal group.

The average score of OSDI of the dry eye group was 27.02 ± 14.35, and the average score of corneal fluorescein in the dry eye group was 3.51 ± 1.67. The average score of corneal fluorescein in the normal group was 7.29 ± 3.36 and the average score of corneal fluorescein in the normal group was 1.23 ± 2.32. These two indicators were significantly higher in the dry eye group than in the normal group (*P* < 0.001). The average of NIBUT in the dry eye group was 6.32 ± 2.49 and was significantly lower than that of the normal group, which was 13.14 ± 3.67 (*P* < 0.001).

### 3.3. Comparison of Meibomian Gland Indexes between the Dry Eye Group and the Normal Group

In contrast with the normal group, the meibomian gland orifice scores, meibomian gland secretion scores, and meibomian gland dropout scores were significantly higher in the dry eye group (*P* < 0.0001) (Table 2).

### 3.4. Correlation Analyses between Scores of Complaining of Dry Eye and Ocular Surface Analysis Indicators

**Figure 1:**
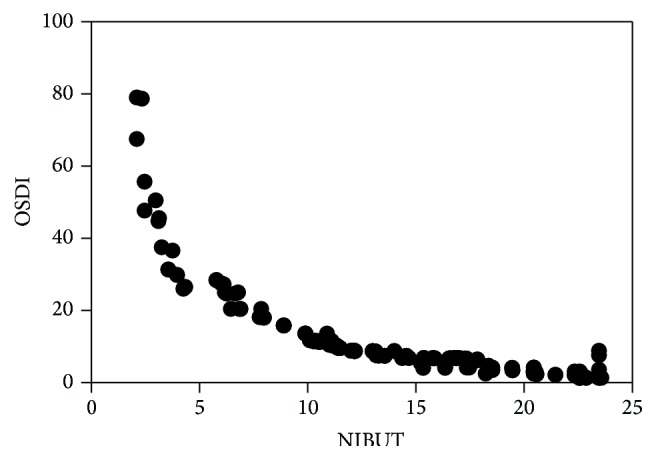
Correlation analysis between NIBUT and OSDI. Negative correlation was found between NIBUT and OSDI in the two groups.

**Figure 2:**
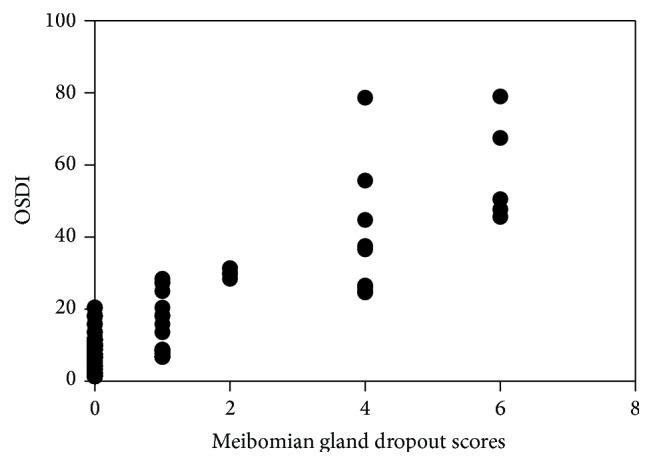
Correlation analysis between meibomian gland dropout scores and OSDI. Positive correlation was found between meibomian gland dropout scores and OSDI in the two groups.

A highly significant inverse correlation was observed between the value of OSDI and NIBUT (rs = −0.982, *P* = 0.000) (Figure 1). Moreover, a highly significant correlation was observed between the value of OSDI and meibomian gland dropout scores (rs = 0.838, *P* = 0.000) (Figure 2).

## 4. Discussion

Recent studies showed that dry eye is a major clinical problem affecting quality of life [4] as it reduces the immunity of ocular surface, causes eye symptoms in children, leads to visual fluctuations during the day, and affects visual clarity in the daytime. Moreover, dry eye can reduce learning efficiency in children. Dry eye is widely believed to be a type of disease whose incidence increases with age [5], and thus scholars have conducted much dry eye research for the elderly. The ability of children to express eye symptoms are worse than adults, or some children may be able to express it clearly but dry eye examinations are difficult. Moreover, allergic conjunctivitis has a higher prevalence in children, and many children who have this condition also suffer from dry eye, making dry eye diagnosis more difficult [16]. Thus, the dry eye incidence in children was underestimated by many scholars. In this study, we use Keratograph 5M combined with slit-lamp examination and dry eye questionnaire to give myopic adolescents a series of dry eye-related inspections and assessments. Dry eye incidence in children was found to be 18.95% which is lower than that in adults but still not significant. Undiagnosed dry eye can lead to fragile ocular surface environment, irreversible eye damage, and increased possibility of corneal ulcers and scars [5]. Accurate diagnosis, systemic treatment, and etiological control can improve eye health and ensure good visual quality in young people.

Keratograph 5M is an objective, comprehensive, and noninvasive dry eye diagnostic device that can detect NIBUT, noninvasive tear meniscus height, and meibomian gland dropout. Keratograph 5M exhibits high accuracy in the dry eye diagnosis in adults [17]. The current study shows that Keratograph 5M has a good implementation even in children, and it can be combined with questionnaire to facilitate clinical diagnosis of dry eye in children. OSDI, NIBUT, and meibomian gland dropout are correlated to dry eye in adolescents, which means that aggravated dry eye symptoms are associated with worse unstable tear film and increased meibomian gland dropout. The lower prevalence of dry eye disease in children relative to adults, limitations of diagnosis, lower degree of the subjective assessment of symptoms in children, and the lack of clinician attention reduce dry eye awareness.

The meibomian glands are the main source of lipids for human tear film. The lipid layer of the tear film slows evaporation of the aqueous of tear film, preserves a clear optical surface, and forms a barrier to protect the eye from microbial agents and organic matter [18]. The meibomian gland plays a more important role than aqueous tear volume in determining the severity of ocular discomfort and dry eye conditions [19]. Lipid-deficient dry eye caused by meibomian gland dysfunction (MGD) has increasingly drawn ophthalmologists' attention. MGD is a chronic, diffuse abnormality of the meibomian glands, commonly characterized by terminal duct obstruction or qualitative/quantitative changes in the glandular secretions. MGD may result in alteration of the tear film, symptoms of eye irritation, clinically apparent inflammation, and ocular surface disease [20]. MGD could reduce tear film stability and cause ocular complaints, inflammation, and other ocular surface disorders [21]. The mean values of tear meniscus height in the dry eye and the normal groups were both in the normal range, whereas NIBUT in the dry eye group was shorter than that of the normal group, which suggests that the dry eye group has normal tear volume but relatively unstable tear film relative to the normal group. The dry eye group of myopic teenagers has a high corneal staining score, more abnormality of meibomian gland orifices and meibomian gland lipid secretions, and more meibomian gland dropouts, causing serious MGD. This result is similar to that of previous studies where lack of meibomian gland is also accompanied by damaged meibomian gland function [7]. This result implies that the common type of dry eye among myopic teenagers is lipid abnormalities of dry eye (i.e., evaporative dry eye). Currently, the clinical evaluation of dry eye is mainly based on BUT and Schirmer tests, whereas the evaluation of meibomian gland function and lipid layer is deficiency. Keratograph 5M, which has a high compatibility in children, has been found to provide early diagnostic and therapeutic values in children for the diagnosis of meibomian gland function and tear film stability. Combined with the questionnaire, the ratio of failure diagnosis of dry eye in children can be reduced.

Currently, the main correction methods of juvenile myopia are frame glasses, contact lens, and orthokeratology (ortho-k). The effectiveness of overnight orthokeratology in flattening the cornea and temporarily reducing myopia has been widely documented [22]. Parents increasingly choose night-wear ortho-k to control myopia of their children. Given that ortho-k is placed on the cornea for the whole night, the ocular surface condition of adolescents with refractive errors should be fully assessed. When considering adolescent ortho-k treatment, we should also pay attention to the situation of the ocular surface of the patients, especially meibomian gland function and dry eye prevalence, which can help improve the safety of the treatment.

The clinical and epidemiological aspects of dry eye in children have not been as well described as in adults [5]. The prevalence of dry eye disease in children varies greatly depending on which criteria and methods were used in previous research. Reportedly, 9.7% of all children have been diagnosed with dry eye disease [4]. Dry eye disease associated with longtime reading can have many signs and symptoms involved, a lot of which are still not understood. Many Chinese children with arduous learning tasks have experienced these signs and symptoms. Myopia has been associated with strenuous near task as well. Blink rates during near work are decreased leading to improper tear film placement. In this study, only normal myopic adolescents were chosen to analyze dry eye and ocular surface. The results suggest that the prevalence of dry eye in adolescents with myopia is 18.95% higher than other research documents entail. For further study regarding dry eye disease in children expanding the number of patients and the inclusion of emmetropes adolescents should be considered.

## Figures and Tables

**Table 1:** Comparison of general condition and ocular surface parameters between the dry eye group and the normal group.

Group	Dry eye	Normal	*P*
Age (year)	12.45 ± 1.54	11.75 ± 1.95	0.051
Sex ratio (male/female)	25/22	98/103	0.175
OSDI	27.02 ± 14.35	7.29 ± 3.36	<0.001
Tear meniscus height (mm)	0.23 ± 0.03	0.22 ± 0.03	0.214
NIBUT (s)	6.32 ± 2.49	13.14 ± 3.67	<0.001
Corneal fluorescein scores	3.51 ± 1.67	1.23 ± 2.32	<0.0001

**Table 2:** Comparison of meibomian gland functional indexes between the dry eye group and the normal group.

Group	Dry eye	Normal	*P*
Meibomian gland orifice scores	1.82 ± 0.53	0.51 ± 0.62	<0.0001
Meibomian gland secretion scores	1.35 ± 0.59	0.41 ± 0.35	<0.0001
Meibomian gland dropout scores	3.21 ± 1.02	0.61 ± 0.65	<0.0001
